# South African hearing conservation programmes in the context of tele-audiology: A scoping review

**DOI:** 10.4102/sajcd.v67i2.670

**Published:** 2020-03-03

**Authors:** Katijah Khoza-Shangase, Nomfundo Moroe

**Affiliations:** 1 Department of Audiology, Faculty of Humanities, University of the Witwatersrand, Johannesburg, South Africa

**Keywords:** audiology availability, e-health, e-medicine, e-practice, hearing conservation, noise, occupational, resource constrained, tele-audiology, tele-health, tele-medicine

## Abstract

**Background:**

The limited involvement of audiologists in occupational noise-induced hearing loss (ONIHL) management through hearing conservation programmes (HCPs) is a global issue. In low- and middle-income (LAMI) countries such as South Africa, this is also exacerbated by demand versus capacity challenges. Tele-audiology is an option requiring serious deliberation by the audiology community within HCPs in LAMI contexts.

**Objectives:**

This scoping review explores if tele-audiology has a potential value in HCPs and reviews what has been documented in the literature on the use of tele-audiology in HCPs.

**Method:**

A scoping review was conducted using the Arksey and O’Malley’s framework. A search was conducted in five electronic bibliographic databases including Science Direct, PubMed, Scopus Medline, ProQuest and Google Scholar and the grey literature to identify publications presenting considerations around tele-audiology in the implementation of HCPs.

**Results:**

Findings revealed significant dearth of evidence specific to the use or application of tele-audiology in ONIHL and/or HCPs both within the African context and internationally, despite the purported potential benefit of this service delivery model, particularly in resource-constrained contexts such as LAMI countries. Of the publications deemed potentially relevant to this scoping review, none were found that specifically investigated or addressed the use of tele-audiology in ONIHL or HCPs as their main objective. Nuanced analysis of publications revealed that in the last decade, indication for potential growth in the use of tele-audiology within occupational audiology is indicated.

**Conclusion:**

Because of the significant demand versus capacity challenges in LAMI countries, and because of the need for scaling up audiology professionals’ management of HCPs, careful consideration of teleaudiology as a platform to deliver services in these contexts is required.

## Introduction

Employees in the mining and several other industries contract various occupational health medical conditions, with occupational noise-induced hearing loss (ONIHL) (reviewed in Rawool, 2011) being one among them (Lie, Skogstad, Johnsen, Engdahl, & Tambs, 2015). This condition presents as a permanent sensorineural hearing loss as a consequence of exposure to hazardous or excessive levels of noise during the performance of one’s occupational duties (Nelson, Nelson, Concha-Barrientos, & Fingerhut, 2005; Thorne, 2006). This hearing loss may be disabling in nature and is symbolised by hearing thresholds below 40 dBs (Yadav, Yadav, Netterwala, Khan, & Desai, 2015). However, other criteria have been used to determine disabling hearing loss including 25 dBHL (Rawool, 2018) mainly because of individual variability in factors such as activity limitations and participation restriction (Olusanya, Neumann, & Saunders, 2014).

Dugan (2003, p. 3) asserts that disabling hearing loss is the ‘most prevalent, least recognised and least understood physical disability’. This view is supported by Copley and Frederichs (2010) as well as Hermanus (2007) who argue that permanent disabling hearing loss is a major contributor to the global burden of disease for individuals, families, communities and countries. Globally, ONIHL has been placed number one on the list of work-related disabilities and is the second most common form of acquired hearing loss after presbycusis (Mostaghaci et al., 2013; Ritzel & McCrary-Quarles, 2008). Nandi and Dhatrak (2008) predicted that ONIHL will gain more prominence as an important public health priority because of populations living longer and the spread of industrialisation, thereby adding to the global burden of this affliction. Within the South African context, the high burden of communicable diseases such as HIV, AIDS and TB within the mining population, which has been found to impact negatively on the severity of ONIHL, significantly adds to this public health challenge (Khoza-Shangase, 2019a).

Acquiring hearing loss in the workplace has significant implications for employees’ health, safety, career sustaining and advancement, job performance and overall quality of life (Amjad-Sardrudi, Dormohammadi, Golmohammadi, & Poorolajal, 2012; Hong, Kerr, Poling, & Dhar, 2013; Kane-Berman, 2017; Picard et al., 2008; Thorne, 2006), with adverse consequences for the economic outlook of both the employers and the employees (Chadambuka, Mususa, & Muteti, 2013; Rikhardsson, 2004; Yongbing & Martin, 2013). These authors argue that the effects of incurring hearing loss in the workplace have adverse consequences on the health, safety and economic outlook of the affected individuals, their families, societies and the state as well; Yongbing and Martin (2013) declare that ONIHL is a potentially costly public health issue, especially in the low- and middle-income (LAMI) countries.

Developing an ONIHL minimises possibilities and opportunities for further employment (Kane-Berman, 2017), and therefore, it negatively impacts the career stability and advancement. A hearing loss, even a mild one, may have a profound impact on the safety and work-related injuries for the affected employee (Amjad-Sardrudi et al., 2012) because the employee’s ability to perform or complete tasks significantly depends on auditory signals or verbal communication (Thorne, 2006). Such a scenario consequently results in a communication handicap, which impacts teamwork and group productivity. Additionally, prolonged exposure to hazardous noise in the workplace can potentially lead to increased fatigue and decreased concentration, leading to increased human errors (Amjad-Sardrudi et al., 2012; Picard et al., 2008) and increased health and safety risks. All these present significant quality-of-life challenges for the employee (Moroe & Khoza-Shangase, 2018; Moroe, Khoza-Shangase, Madahana, & Nyandoro, 2019).

Financially, Hong et al. (2013) argue that although the impact of ONIHL on one’s health and quality of life cannot be quantified in tangible measures or standards, compensation costs for incidents of ONIHL are consistently increasing. As an illustration, the approximated total cost for occupational accidents and diseases ranges between 1% and 3% of the gross domestic product (GDP) in various countries (Rikhardsson, 2004). This is high for LAMI countries, although exact statistics on the burden of ONIHL in these countries are not readily available (Nelson et al., 2005). Chadambuka et al. (2013, p. 899), nonetheless, argue that 80% of individuals affected by ONIHL reside in LAMI countries where ONIHL presents a ‘much heavier burden than in developed regions of the world’. It is for this reason ONIHL is considered one of the greatest threats to a country’s economy as well as its public health.

In South Africa, the mining industry is one of the influential backbones supporting the country’s political and economic growth. Economically, mining contributes a hugely significant amount, R286 billion, towards South Africa’s GDP. Furthermore, this sector is considered a major employer, producing 4.5 million jobs, and contributes R10bn to the fiscus annually through pay as you earn (PAYE) (Teke, 2017). Occupational noise-induced hearing loss, therefore, has an impact beyond the individual affected, and hence the importance of hearing conservation programmes (HCPs).

Despite the implementation of HCPs, which are aimed at protecting workers with significant occupational noise exposures (Occupational Safety and Health Administration – OSHA, 2002), ONIHL remains a prevalent condition in the South African mining industry (Edwards, Dekker, Franz, Van Dyk, & Banyini, 2011; Kanji, Khoza-Shangase, & Ntlhakana, 2019; Khoza-Shangase, 2019b; Ntlhakana, Kanji, & Khoza-Shangase, 2015; Strauss, Swanepoel, Becker, Eloff, & Hall, 2012; Van Coller, 2015). In developed countries, the incidence of ONIHL has been documented to be decreasing or at least stabilising because of advances in technology that have seen these countries investing in quieter equipment and machinery in industries prone to excessive noise (Morata & Meinke, 2016; Safe Work Australia, 2010). *Buying quiet* is part of successful HCPs, which the South African Mine Health and Safety Council (MHSC) aspires towards.

The South African MHSC states one of its goals as ‘every mine worker returning from work unharmed everyday: Striving for zero harm’ (MHSC, 2015). As far as ear and hearing health are concerned, this goal has not been realised despite the concerted efforts of the MHSC and the Chamber of Mines in South Africa (Booyens, 2013). The reality is, approximately 73.2% of miners in South Africa are exposed to excessive noise surpassing the legislated occupational exposure limit of 85 dBs, despite HCPs being implemented in the mining sector (Edwards et al., 2011; Strauss et al., 2012), with numerous evidence from this context indicating little, if any, success of HCPs (Edwards et al., 2011; Edwards, Milanzi, Khoza, Letsoalo, & Zungu, 2015; Edwards & Kritzinger, 2012; Kanji, Khoza-Shangase, & Ntlhakana, 2019; Moroe & Khoza-Shangase, 2018, 2019b; Moroe, Khoza-Shangase, Kanji, & Ntlhakana, 2018; Ntlhakana et al., 2015; Strauss et al., 2012).

Literature suggests that the success of HCPs is heavily dependent on adhering to all HCP pillars (Amedofu, 2007; Hong et al., 2013). These pillars include periodic noise exposure measurement and monitoring; engineering controls; administrative controls; personal hearing protection; employee and management education, motivation and training; risk-based medical examination; medical surveillance and audiometric evaluations, as well as record keeping (Amedofu, 2007; Hong et al., 2013). These fall within the scope of audiology, although the role of audiologists in the mining industry has not received much attention, with evidence from the South African context indicating very limited involvement of audiologists in HCPs. Moroe and Khoza-Shangase (2018) found that the small numbers of audiologists within this field in South Africa experience scope-context misalignment, juniorisation as experts, questions about their role and value, as well as limited training in occupational audiology. These authors conclude that evidence from their study highlights important gaps in HCPs in South Africa, with audiologists being only minimally and peripherally involved in the management of ONIHL in the South African mining sector. This minimal involvement of audiologist is arguably because of the fact that the South African Department of Labour regulations into ONIHL minimise the role of audiologists with prominence given to Occupational Health Officers and Otorhinolaryngologists (Department of Labour, 2019). This is despite Health Professions Council of South Africa (HPCSA, 2012) regulations and the American Academy of Audiology (1997) providing guidance that audiologists should be the principal advocates for and the supervisors of HCPs.

A profession’s ability to fulfil its functions and mandate in any context is influenced by the availability of resources. South Africa has significant challenges when it comes to manpower resources, and these challenges negatively influence service provision nationally, with the mining sector also being affected. Demand versus capacity rages on as a single big barrier to the provision of health care, particularly in the public sector which caters for approximately 80% of the South African population. (Khoza-Shangase, 2019a). This demand versus capacity challenge becomes far worse in contexts that are not traditional spaces of practice for audiologists in South Africa, such as occupational audiology contexts. For a population of over 55 million, HPCSA records reflected negligible numbers of speech language and hearing (SLH) professionals registered with the HPCSA by October 2018. Specifically, there were only 1589 speech therapists and audiologists, 642 audiologists and 157 hearing aid acousticians for the entire South African population – servicing both the public and private sectors. The audiologists’ numbers are worse in the four main provinces where mining is the largest industry – North West (8), Limpopo (17), Mpumalanga (38) and Northern Cape (7). These numbers show the demand versus capacity challenge clearly in the provision of SLH services in the country (Khoza-Shangase, 2019a), with obvious implications for the implementation and monitoring of HCPs where required. This highlights the importance of exploring tele-audiology within this context.

Tele-audiology has been defined as a subset of tele-health that has its goal as increasing access to audiological services primarily in areas with limited access to health care because of shortage of resources, as in the South African context and, for the current paper, more so in the occupational audiology sector. Telecommunication technologies are utilised in order to access patients, reduce barriers to optimal care in underserved areas, improve user satisfaction and accessibility to specialists, decrease professional isolation in rural areas, help medical practitioners expand their practice reach and save patients from having to travel or be transported to receive high-quality care (Krupinski, 2015). There are obvious advantages of tele-audiology in the South African occupational health context requiring deliberation, hence the rationale for current scoping review.

## Methods

This scoping review commenced as a systematic review; however, because of dearth of sufficient evidence to allow for this type of review, it was changed to a scoping review, where the first step was the establishment of a research team consisting of two individuals with expertise in ONIHL, HCPs and research synthesis, as advocated by Levac, Colquhoun and O’Brien (2010). The team advised on the broad research question to be addressed and the overall study protocol, including identification of search terms and selection of databases to search.

The researchers adopted Arksey and O’Malley’s (2005) framework while adhering to recommendations by Levac et al. (2010). The review included the following five key phases: (1) identifying the research question, (2) identifying relevant publications, (3) study selection, (4) charting the data and (5) collating, summarising and reporting the results (Arksey & O’Malley, 2005).

### Research question

The current review was directed by the following question: ‘does tele-audiology have a potential value in HCPs and what has been documented in the literature on the use of tele-audiology in HCPs?’ This question was guided by the prevailing demand versus capacity challenges in LAMI countries, and the researchers wanted to conduct this review in order to synthesise available evidence so as to map the literature on tele-audiology and HCPs and gain access to an opportunity to identify gaps in the evidence, and types and sources of evidence to inform training, practice, policy-making and research– as advised by Daudt, Van Mossel and Scott (2013) on the value of scoping reviews.

### Data sources and search strategy

The initial search was implemented on 27 March 2019, in six electronic databases: Science Direct, PubMed, Scopus Medline, ProQuest and Google Scholar. The databases were selected to be comprehensive and to cover the use of tele-audiology in HCPs. The selected studies were restricted to studies published in English with a focus in tele-audiology. The search query consisted of the following terms: e-health, e-medicine, e-practice, tele-health, tele-audiology, tele-medicine, industry, occupational, hearing conservation, resource constrained and audiology availability were considered by the authors to describe the scoping review and its methodology.

Applying the same search process or string that was used for the search in Science Direct, PubMed, Scopus Medline and ProQuest, a web search was conducted in Google Scholar to identify grey literature. The a priori decision was made to screen only the first 10 hits (as sorted by relevance by Google Scholar) after considering the time required to screen each hit and because it was believed that further screening was unlikely to yield many more relevant articles (Stevinson & Lawlor, 2004). The following websites were also searched manually: The MHSC, Minerals Council South Africa, Department of Mineral Resources and Occupational Health and Safety websites. Furthermore, the reference list of all the screened articles was also manually searched for further articles not yet captured. The following citations were included: Audiology Australia (2015), Norris et al. (n.d.), Meinke et al. (2017), Brennan-Jones, Eikelboom and Swanepoel (n.d.); Swanepoel et al. (2010), Swanepoel, Matthysen, Eikelboom, Clark and Hall (2015) and MacLennan-Smith, Swanepoel and Hall (2013). Snowball sampling was adopted where citations meeting the inclusion criteria within publications were searched. A subsequent search of the above-mentioned bibliographic database and grey literature was conducted again in August 2019 to ascertain if there were additional publications post the initial search. One new hit, Dietz (2019), was identified.

### Citation management

All citations were imported into the web-based bibliographic manager Endnote. Duplicate citations were removed manually with further duplicates removed when found later in the process for subsequent title and abstract relevance screening and data characterisation of full articles.

### Eligibility criteria

A two-stage screening process was used to assess the relevance of publications identified in the search. Publications were eligible for inclusion if they contained the keywords and phrases, and if they broadly described the use of tele-audiology in ONIHL and/or HCPs in order to determine and characterise the existing evidence based on the use of tele-audiology in HCPs. Because of limited resources for translation, only evidence published in English were included. Publications that described tele-audiology without mentioning HCPs and/or ONIHL assessment and management were excluded from the analysis.

### Title and abstract relevance screening

For the first level of inspection, only the title and abstract of citations were reviewed for efficient time management – where resources could be wasted by obtaining articles that did not meet the minimum inclusion criteria for this coping review. The researchers used a title and abstract relevance screening spreadsheet that they developed. The spreadsheet had previously been used by the same research team to evaluate reviewer agreement, where the overall kappa of the pretest was >0.8 which is considered to represent a high level of agreement (Viera & Garret, 2005). The title and abstract of each citation were independently screened by both researchers. Titles for which an abstract was not available were included for subsequent review of the full article, which was done for all publications, in the data characterisation phase. The researchers met regularly during this process to ensure that any conflicts were resolved, with the lead author making the final decision where disagreements were present. A high level of agreement was found with the overall kappa at 0.83.

### Data characterisation

All full citations that were found to be relevant for the current scoping review on tele-audiology and HCPs after title and abstract inspection were extracted for later review of the full-text article or document. A spreadsheet was developed by the authors where relevance of the publication was confirmed and where details of the publication such as type of publication, author and publication year, title, focus and aims, methodology, context, results and/or recommendations and reported challenges, gaps and limitations were recorded. The characteristics of each publication were extracted by both authors. Further publications were excluded at this phase if they did not meet the minimum eligibility criteria. In line with Levac et al. (2010), following independent reviews of all the publications, the authors met to resolve any conflicts and ensure consistency between them as well to make sure that the publications were consistent with the set research question and purpose.

### Data summary and synthesis

**FIGURE 1:**
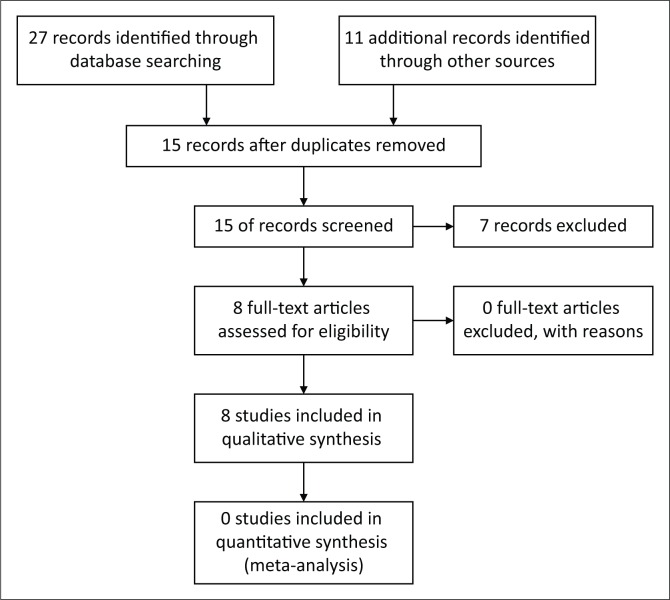
Preferred Reporting Items for Systematic Reviews and Meta-Analysis (PRISMA) flow diagram for included publications.

The data were compiled in a single spreadsheet and imported into Microsoft Excel 2016 (Microsoft Corporation, Redmond, WA) for descriptive analysis (Figure 1).

### Ethical considerations

This article followed all ethical standards for a research without direct contact with human or animal subjects.

## Results and discussion

The research question for this scoping review was as follows: ‘does tele-audiology have a potential value in HCPs and what has been documented in the literature on the use of tele-audiology in HCPs?’

Findings revealed significant dearth of evidence on the use or application of tele-audiology in ONIHL and/or HCPs both within the African context and internationally, despite the purported potential benefit of this service delivery model, particularly in resource-constrained contexts such as LAMI countries (Audiology Australia, 2015; Brennan-Jones et al., n.d.; Krupinski, 2015; Swanepoel et al., 2010). Of the publications deemed potentially relevant to this scoping review, not a single publication was found that specifically investigated or addressed the use of tele-audiology in ONIHL or HCPs.

Nuanced analysis of publications revealed that in the last decade, evidence of tele-audiology in general audiology practice is increasing, with indication for possible growth in the use of this service delivery model within occupational audiology, as concluded by the current authors. This potential increase in tele-audiology use within this field is indicated by studies such as those investigating the following aspects which the current authors believe have implications for tele-audiology in ONIHL and its management (Table 1): the use of mobile technology for booth-less audiometry; use of wireless and booth-less technology for hearing testing in industry; conducting pure-tone audiometry outside a sound booth using earphone attenuation, integrated noise monitoring and automation; validity of diagnostic pure-tone audiometry without a sound-treated environment and with position papers and clinical guidelines on the need, use and potential benefits of tele-audiology in under-served communities. All these publications indirectly speak to the audiometric evaluations pillar of HCPs with no evidence found looking at the other pillars such as periodic noise exposure measurement and monitoring; engineering controls; administrative controls; personal hearing protection; employee and management education, motivation and training; risk-based medical examination and medical surveillance, as well as record keeping, all of which the current authors believe are amenable to tele-audiology.

**TABLE 1:** Studies with implications for tele-audiology in occupational noise-induced hearing loss and its management.

Author(s) (date)	Publication title	Publication focus and aims	Methodology	Context	Results
Audiology Australia (2015)	Submission to the MBS Review Taskforce Consultation Paper	Audiology Australia provides to the Medicare Benefits Schedule Review Taskforce (the Taskforce), a submission in response to the Consultation Paper, Public Submissions (September 2015)	Position paper	Australia	Audiology Australia updates the MBS on current tele-practice models of audiological service delivery. *Rules and Regulations Requiring that Audiological Services must be Provided in Person* highlight new and exciting opportunities for the delivery of audiological services, especially in remote areas tele-audiology and tele-otology
Norris et al. (n.d.)	Mobile technology for booth-less audiometry	Conducting audiometry using internet technologies is difficult because ambient noise can affect the results		USA	This paper presents two human studies evaluating a prototype noise attenuating, wireless audiometric headset that pairs with a mobile device to administer automated audiograms
Meinke et al. (2017)	Going wireless and booth-less for hearing testing in the industry	To assess the test–retest variability of hearing thresholds obtained with an innovative, mobile WAHTS with enhanced sound attenuation to test industrial workers at a worksite as compared to standardised automated hearing thresholds obtained in a mobile trailer sound booth	A within-subject repeated-measures design was used to compare air-conducted threshold tests (500–8000Hz) measured with the WAHTS in six workplace locations, and a third test using computer-controlled audiometry obtained in a mobile trailer sound booth. Ambient noise levels were measured in all test environments. Study sample: 20 workers served as listeners and 20 workers served as operators	USA	The WAHTS resulted in equivalent thresholds as the mobile trailer audiometry at 1000, 2000, 3000 and 8000Hz and thresholds were within±5dB at 500, 4000 and 6000Hz
Brennan-Jones et al. (n.d.)	Tele-health For diagnosis of hearing loss : Open access guide to audiology and hearing aids for otolaryngologists	This chapter describes some of the options available and the benefits and limitations of tele-health	-	-	-
Swanepoel et al. (2010)	Tele-health in audiology: The need and potential to reach underserved communities	Review paper on tele-audiology	-	-	Although many questions related to aspects such as quality control, licensure, jurisdictional responsibility, certification and reimbursement still need to be addressed, no alternative strategy can currently offer the same potential reach for impacting the global burden of hearing loss in the near and foreseeable future
Swanepoel et al. (2015)	Pure-tone audiometry outside a sound booth using earphone attenuation, integrated noise monitoring, and automation	This study investigated the validity of an automated mobile diagnostic audiometer with increased attenuation and real-time noise monitoring for clinical testing outside a sound booth	Attenuation characteristics and reference ambient noise levels for the computer-based audiometer (KUDUwave) was evaluated alongside the validity of environmental noise monitoring. Clinical validity was determined by comparing air- and bone-conduction thresholds obtained inside and outside the sound booth (23 subjects). Study sample: 23 normal-hearing subjects (age range, 20–75 years; average age 35.5) and a sub group of 11 subjects to establish test–retest reliability	South Africa	Improved passive attenuation and valid environmental noise monitoring was demonstrated. Clinically, air-conduction thresholds inside and outside the sound booth, corresponded within 5 dB or less. Bone conduction thresholds corresponded within 5 dB or less in 80% of comparisons between test environments. Threshold differences were not statistically significant. Mean absolute test–retest differences outside the sound booth was similar to those in the booth
MacLennan-Smith et al. (2013)	Validity of diagnostic pure-tone audiometry without a sound-treated environment in older adults	To investigate the validity of diagnostic pure-tone audiometry in a natural environment using a computer-operated audiometer with insert earphones covered by circumaural earcups incorporating real-time monitoring of environmental noise	A within-subject repeated measures design was employed to compare air (250 to 8000 Hz) and bone (250 to 4000 Hz) conduction pure-tone thresholds, measured in retirement facilities, with thresholds measured in a sound-treated booth. Study sample: 147 adults (average age 76 ± 5.7yrs) were evaluated. Pure-tone averages were 25 dB in 59%, mildly (40 dB) elevated in 23%, and moderately (55 dB) elevated in 6% of ears	South Africa	Air-conduction thresholds (*n* = 2259) corresponded within 0 to 5 dB in 95% of all comparisons between the two test environments. Bone-conduction thresholds (*n* = 1669) corresponded within 0 to 5 dB in 86% of comparisons. Average threshold differences (0.6–1.1) and standard deviations (3.3–5.9) were within typical test–retest reliability limits. Thresholds recorded showed no statistically significant differences
Dietz (2019)	The future of hearing conservation	Update or letter	Deliberation on technological innovations around smart phones, tablets, and the mobile Internet – which has led to commercially available tablet audiometers		Possible future use of these in HC, but with cautions around validation, sensitivity, and specificity of these measures

MBS, Medicare Benefits Schedule; WAHTS, wireless automated hearing-test system; HC, hearing conservation.

As far as the use of mobile technology for booth-less audiometry is concerned, current evidence indicates that the use of technologies such as Internet is possible, as long as ambient noise can be efficiently managed (Norris et al., n.d.), and hence the importance of studies investigating booth-less audiometry. This is particularly important as accessibility of audiometry has been argued to be significantly impeded by the cost of sound booths (Brennan-Jones et al., n.d.), over and above the shortage of hearing health personnel in the South African context (Moroe & Khoza-Shangase, 2018). Norris et al. (n.d.), from their two studies evaluating a prototype noise attenuating, wireless audiometric headset that pairs with a mobile device to administer automated audiograms, had positive outcomes which proved that conducting audiometry using Internet technologies is possible if ambient noise is controlled.

Norris et al.’s (n.d.) findings are consistent with their recent findings (Meinke, Norris, Flynn, & Clavier, 2017) on the use of wireless and booth-less technology for hearing testing in industry, which found comparable performance with the use of innovative, mobile wireless automated hearing test system (WAHTS) in occupational audiometry and valid thresholds in diverse test locations without the use of sound-attenuating enclosures. This study focused on the test–retest variability of hearing thresholds obtained with WAHTS with enhanced sound attenuation to test industrial workers at a worksite as compared to standardised automated hearing thresholds obtained in a mobile trailer sound booth. These findings have significant implications for the audiometric evaluation pillar of HCPs.

Additionally, Maclennan-Smith et al.’s (2013) research on the validity of diagnostic pure-tone audiometry without a sound-treated environment indicates that valid diagnostic findings can be obtained in a natural environment with recently developed technology, again offering the possibility of access to diagnostic audiometry in communities such as the South African occupational audiometry context where sound-treated booths may not be available. The automated aspect of this study as it investigated the validity of an automated mobile diagnostic audiometer with increased attenuation and real-time noise monitoring for clinical testing outside a sound booth has direct implications for the tele-HCPs’ audiometric evaluation pillar. In their study, these authors found that air-conduction thresholds corresponded within 0 to 5 dB in 95% of all comparisons between the two test environments, and bone-conduction thresholds did the same in 86% of comparisons. When assessing the validity of an automated mobile diagnostic audiometer during conduction of pure-tone audiometry outside a sound booth using earphone attenuation, integrated noise monitoring and automation, Swanepoel et al. (2015) also demonstrated reliable hearing assessments. Dietz (2019) raises cautions about validation, sensitivity and specificity in his deliberations on technological innovations around smartphones, tablets and the mobile Internet – which has led to commercially available tablet audiometers seen as the future in hearing conservation.

Such evidence is supported by recommended clinical guidelines and position papers that guide the profession of audiology with tele-audiology use, although the evidence is restricted to only one pillar of HCPs. These position papers and guidelines do not specifically speak to the context of occupational audiology, a context which comprises of several factors that require consideration. These factors include (1) the nature of the service required (HCPs with specific pillars), (2) demand versus capacity challenges with regard to availability of audiologists practicing within this area and (3) litigation risks linked to occupational injury claims for ONIHL, therefore the importance of valid and reliable measures that can not only guide clinical management but can also stand up to the scrutiny of the courts.

Audiology Australia (2015) provides a response to the Medicare Benefits Schedule (MBS) Review Taskforce’s Consultation Paper, where they argue for inclusion of tele-audiology as it is likely to shape future audiological practice by changing the way services are delivered to remote areas of Australia where populations have limited access to health services. Audiology Australia’s position is that tele-practice is an appropriate model of service delivery for the audiology profession. The implication of this position paper for the current scoping review and the possible implementation of tele-audiology within occupational audiology in the South African context is that of medical insurance cover – an issue that South African health care has not addressed in both practice and regulations. Swanepoel et al. (2010) cautioned about reimbursement questions surrounding tele-audiology when they listed challenges that still require attention including aspects such as quality control, licensure, jurisdictional responsibility and certification.

Within the South African context, Siegfried, Wilkinson and Hofman (2017) report that South Africa has not made significant strides in its progress for health technology assessment (HTA). A legal and policy landscape analysis reveals that no specific provision in the National Health Act exists, and HTA is narrowly and incompletely defined (Siegfried et al., 2017). Siegfried and colleagues take this into account and call for the National Department of Health to host an HTA summit in order to gain consensus on an acceptable and useful definition of HTA appropriate to the South African context and to deliberate on the policy and legislative requirements for a national HTA agency or alternative mechanism in South Africa. These authors also suggest that consideration should be given to the revision of relevant national legislation and policy in order to align with the imminent National Health Insurance (NHI) that has universal health coverage as a goal, and the current authors believe that this is the opportune time for the audiology community to also advance considerations around tele-audiology as a service delivery model in occupational audiology. It is hoped that resolutions from this summit will positively impact tele-audiology in HCPs agenda and general developments in the field of HTA within the South African context. This positive impact might extend to include audiologists’ awareness, confidence and their use of tele-audiology, which is currently low.

In Eikelboom and Swanepoel’s (2016) international survey of audiologists’ attitudes towards tele-health, findings indicated positive attitudes towards tele-health and associated technology. Although positive attitude was displayed by respondents, less than a quarter of the participants had actually utilised tele-audiology in their practice. Current authors argue that such findings might actually be worse in LAMI contexts, such as South Africa, where over and above attitudes influencing the use of tele-audiology, access to technology as well as connectivity might place additional impact on the uptake of this service delivery model. These challenges, low numbers of audiologists willing to utilise tele-audiology as well as limited access to technology and connectivity, will hamper all efforts to increase access to audiological services – particularly with the known and documented demand versus capacity quandary within the South African occupational health context.

**FIGURE 2:**
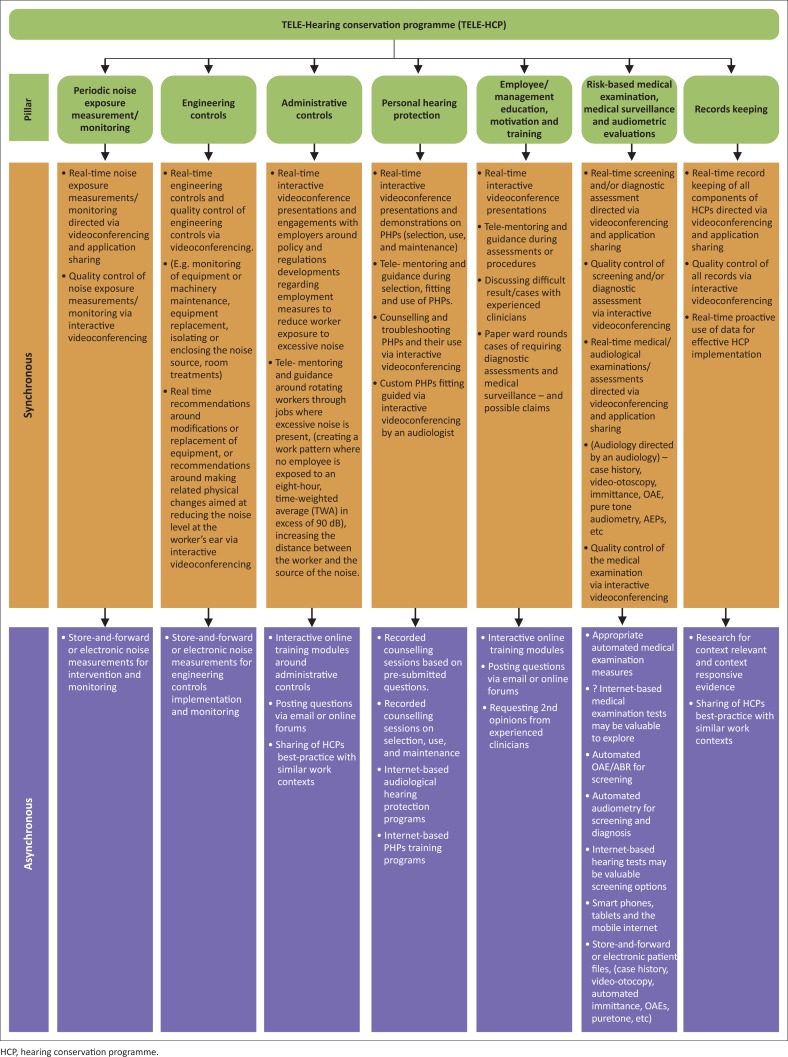
Possibilities of tele-audiology use in hearing conservation programmes for all pillars (tele-hearing conservation programmes).

In reviewing HCPs and all the pillars that comprise them, the current authors strongly suggest that increased strategic efforts be deliberated by the South African audiology community on the use of tele-audiology as an alternative and/or interim measure in delivering the much-required services in this population. The current authors suggest that the South African audiology community should be ready for structured, systematic and sustainable implementation of tele-HCPs once summit resolutions are adopted; their recommended deliberation around the tele-HCP implementation options is depicted in Figure 2.

This deliberation would require careful look at all the HCPs pillars and evidence gathering on how tele-audiology could be used efficiently and ethically in this field, whilst maintaining standards and adhering to regulations and scopes of practice as advocated by the HPCSA and the South African Department of Labour. Maintaining standards and adhering to regulations are particularly important in this field as paraprofessionals, nurses or trained volunteers will be required to facilitate the tele-practice set-up at the industrial site which is remote to the audiologist managing the HCP, and this necessitates the use of recordings, online interactive engagements and/or real-time via interactive videoconferencing. Figure 2 suggests ways in which the pillars of HCPs, including administrative controls, engineering controls, periodic noise exposure monitoring, personal hearing protection, audiometric evaluations, employee and management education and training and record keeping, could all be conducted and managed via tele-audiology. Efficacy of this service delivery model would require future investigation in order to ensure that methods adopted are evidence-based and best practice for the South African context. Investigating other service delivery models such as the United States service model where certified Occupational Hearing Conservationists can provide some hearing conservation services under the indirect supervision of an audiologist or a physician should also be considered.

## Conclusions

The current scoping review clearly indicated lack of evidence on the use of tele-audiology in ONIHL and/or HCPs. This indicates a missed opportunity to increase access to audiological services for this population, particularly with the reviewed evidence of technological advancements that have applicability in the audiometric evaluation pillar of HCPs. Technological advances with their counterparts’ recent advances in tele-health have increased possibilities for alternative service delivery models that are contextually responsive, such as tele-audiology in HCPs. With regard to the audiometric evaluations pillar of HCPs, the introduction of mobile technology for booth-less audiometry; use of wireless and booth-less technology for hearing testing; conduction of pure-tone audiometry outside a sound booth using earphone attenuation, integrated noise monitoring and automation; conduction of diagnostic pure-tone audiometry without a sound-treated environment; and hearing screening using mobile apps are advances that have significantly increased the audiology community’s ability to provide hearing services to remote and resource-limited contexts, such as the occupational setting within the South African context. Expansion of tele-audiology services to the rest of HCPs pillars is a real possibility that has the potential to be productively exploited via both real-time synchronous and asynchronous ‘store-and-forward’ tele-HCPs.

These recommended tele-HCPs must take careful cognisance of policy and regulation challenges, with strict adherence to ethics, human rights and medical law. In their quest to increase access to audiological services within the occupational audiology space where medico-legal claims are a possibility, audiologists must utilise this service delivery model ensuring that the six ethical challenges related to tele-audiology, as presented by Naudé and Bornman (2019), have been resolved and that there are clear guidelines and regulations around them. These six challenges, including licensure, competence, privacy and confidentiality, informed consent, effectiveness of services and program validation, and reimbursement for services, are key for efficient, successful and ethical use of tele-HCPs.

The readers are cautioned to interpret current findings having taken cognisance of identified limitations in this scoping review. Firstly, very few articles were found to have included experimental research to investigate the efficacy of remote audiological monitoring, which led to the recommendation for investigations made by the current authors. Secondly, some of the articles found (e.g. those by Swanepoel) raise questions about conflict of interest or bias because this author is the inventor of the booth-less auditory threshold equipment. Lastly, the challenge of connectivity, which is a significant challenge in LAMI contexts, is silent in the papers reviewed.
